# HIV provider and patient perspectives on the Development of a Health Department “Data to Care” Program: a qualitative study

**DOI:** 10.1186/s12889-016-3152-4

**Published:** 2016-06-10

**Authors:** Julia C. Dombrowski, James W. Carey, Nicole Pitts, Jason Craw, Arin Freeman, Matthew R. Golden, Jeanne Bertolli

**Affiliations:** Department of Medicine, University of Washington, 325 Ninth Ave, Box 359777, Seattle, WA 98104 USA; Public Health – Seattle & King County HIV/STD Program, Seattle, WA USA; Centers for Disease Control and Prevention, Atlanta, GA USA; ICF International, Atlanta, GA USA; Departments of Medicine and Epidemiology, University of Washington, Seattle, WA USA

**Keywords:** Public Health, HIV prevention, HIV care continuum, Qualitative research, Surveillance

## Abstract

**Background:**

U.S. health departments have not historically used HIV surveillance data for disease control interventions with individuals, but advances in HIV treatment and surveillance are changing public health practice. Many U.S. health departments are in the early stages of implementing “Data to Care” programs to assists persons living with HIV (PLWH) with engaging in care, based on information collected for HIV surveillance. Stakeholder engagement is a critical first step for development of these programs. In Seattle-King County, Washington, the health department conducted interviews with HIV medical care providers and PLWH to inform its Data to Care program. This paper describes the key themes of these interviews and traces the evolution of the resulting program.

**Methods:**

Disease intervention specialists conducted individual, semi-structured qualitative interviews with 20 PLWH randomly selected from HIV surveillance who had HIV RNA levels >10,000 copies/mL in 2009–2010. A physician investigator conducted key informant interviews with 15 HIV medical care providers. Investigators analyzed de-identified interview transcripts, developed a codebook of themes, independently coded the interviews, and identified codes used most frequently as well as illustrative quotes for these key themes. We also trace the evolution of the program from 2010 to 2015.

**Results:**

PLWH generally accepted the idea of the health department helping PLWH engage in care, and described how hearing about the treatment experiences of HIV seropositive peers would assist them with engagement in care. Although many physicians were supportive of the Data to Care concept, others expressed concern about potential health department intrusion on patient privacy and the patient-physician relationship. Providers emphasized the need for the health department to coordinate with existing efforts to improve patient engagement. As a result of the interviews, the Data to Care program in Seattle-King County was designed to incorporate an HIV-positive peer component and to ensure coordination with HIV care providers in the process of relinking patients to care.

**Conclusions:**

Health departments can build support for Data to Care efforts by gathering input of key stakeholders, such as HIV medical and social service providers, and coordinating with clinic-based efforts to re-engage patients in care.

## Background

The role of U.S. health departments in HIV prevention has evolved substantially in the past decade. Public health programs are in various states of transition from a traditional HIV prevention model centered on health education, HIV counseling and testing, and behavioral risk reduction, to a contemporary model focused on optimizing the HIV care continuum [1, 2]. Laboratory reporting of CD4 count and HIV RNA [viral load (VL)] results allows health departments to monitor linkage to and retention in HIV care among persons living with HIV (PLWH) and provides an opportunity for health departments to facilitate sustained patient engagement in care [3, 4]. The Centers for Disease Control and Prevention (CDC) encourages health departments to use HIV surveillance data to identify HIV-diagnosed persons who are not in care and intervene to engage or reengage them in care [5, 6].

Data to Care involves a fundamental change in how health departments use HIV surveillance data. Traditionally, many health departments considered these data to be a tool for monitoring the epidemic, not a resource for identifying individual patients for intervention. In contrast, communicable disease programs focused on infections such as syphilis and tuberculosis have used surveillance data to direct public health outreach for decades [7, 8]. The reasons for this HIV exceptionalism include the stigma associated with HIV, the infection’s chronicity, and the lack of a cure [9]. However, HIV is highly treatable and antiretroviral treatment is now a cornerstone of prevention [10]. This reality has prompted a change in how health departments use surveillance data, but instituting that change requires support from key stakeholders, particularly medical providers and the populations affected by HIV [6, 11].

The Public Health – Seattle & King County (PHSKC) HIV/STD Program implemented a Data to Care program in 2011. The Care and Antiretroviral Therapy (ART) Promotion Program (CAPP) is described in detail elsewhere [12]. Briefly, health department Disease Intervention Specialists (DIS) conduct surveillance-based outreach to assist clients in relinking to care through health systems navigation, brief counseling, and referral to support services. To ensure that the program PHSKC planned to develop would meet the needs of PLWH and garner support from medical providers, PHSKC gathered stakeholder input on the Data to Care concept. This effort included in-depth interviews with HIV care providers and PLWH that substantially influenced the development of the program.

The health department in Seattle-King County was an early adopter of the Data to Care paradigm [6, 11, 13]. In the U.S. as a whole, the Data to Care strategy has been implemented heterogeneously to date. Many, perhaps most, state and local health departments are in the early stages of implementing Data to Care programs. Thus, the themes highlighted in the Seattle-King County interviews remain salient today, in part because they focus on the evolving role of the health department in improving patient engagement in HIV care. In order to share insights gained from the interviews that PHSKC conducted during the formative phase of the Data to Care program, PHSKC and CDC investigators collaborated to conduct a formal qualitative analysis of the interviews and we trace the evolution of the program in Seattle-King County. Our findings are particularly relevant for health departments outside of Seattle that are in beginning to develop a Data to Care strategy.

## Methods

### Study design, recruitment and eligibility

Investigators from the University of Washington (UW) and PHSKC DIS staff conducted semi-structured qualitative interviews [14] with 20 PLWH who were not on ART in 2009–2010. Investigators used surveillance data to randomly select persons ≥18 years of age diagnosed with HIV >6 months whose most recently reported viral load was >10,000 copies/mL. The rationale for these criteria was to identify persons with stable HIV diagnoses who were unlikely to be taking ART and would thus be candidates for the potential health department intervention. In order to solicit input from persons for whom the U.S. HIV treatment guidelines (in place at the time of the interviews) recommended different treatment approaches [15], the investigators stratified the random sampling of PLWH by last reported CD4 count (<350, 350–500, and >500 cells/mm^3^).

Beginning in 2009, two DIS contacted the identified persons and asked permission to speak with them by phone about “a public health program.” For patients who gave verbal consent, the DIS offered participation in an in-depth, face-to-face interview for persons with HIV not currently taking ART to guide the development of the program. Persons who had initiated ART in the interim or did not speak English were not eligible for the interview. The UW investigators recruited HIV medical providers for key informant interviews using purposive sampling to solicit input from different types of clinics (e.g., university-affiliated and non-affiliated; public and private), different types of physicians (e.g., physicians and mid-level providers; specialist and primary care doctors), and all major HIV care sites in the area. The goal of this sampling strategy was not to assess differences between providers, but rather to obtain input from diverse perspectives and from key opinion leaders in the community. Prior to the interviews, the investigators planned to obtain input from at least 20 PLWH and 15 medical providers and to conduct additional interviews, if necessary, until saturation was achieved.

### Data collection

The DIS who initially contacted the PLWH participants also conducted the in-depth interviews. A physician investigator conducted the provider interviews. All interviews were conducted in private settings and lasted approximately 45 min. The interviewers used semi-structured interview guides, with separate guides for physicians and patients, to ask participants about how they made decisions regarding ART initiation, their perceptions of barriers to HIV care, and their opinions about the idea of the health department directly contacting PLWH to facilitate care engagement and promote ART. To supplement the qualitative data with descriptive data on respondents, interviewers collected limited self-reported demographic data [gender, race/ethnicity, age, time in practice (providers only), and sexual orientation (patients only)]. All interviews were audio recorded after participants provided verbal consent. PLWH received a financial incentive of $50, and providers received $10 coffee cards.

### Ethics, consent, and permissions

The interviews were conducted for the primary goal of informing development of a local public health program, and thus were not subject to Institutional Review Board (IRB) oversight. The UW IRB approved formal qualitative analysis of the interviews and sharing of de-identified data with CDC investigators. CDC approved the participation of CDC staff in the analysis and authorship of this manuscript.

### Data analysis

The UW/PHSKC investigators sent the de-identified interview transcripts to a research team at CDC for coding and analysis. Four study researchers developed a qualitative interview codebook containing thematic codes, using a standardized iterative process; themes included behaviors, experiences, or opinions reported by the respondents [16]. Differences were resolved through discussion, and coding criteria were modified to accurately reflect the themes present in the transcripts and to ensure that they could be used reliably by all members of the coding team [17]. Four researchers independently coded the interview transcripts (two per interview) using CDC EZ-Text software (version 4.06, CDC, 2013). Agreement between the pairs of coders was assessed, and coding differences were resolved as they arose through discussion and data reevaluation.

## Results and discussion

The demographics of the 20 PLWH who were interviewed are presented in Table 1a. Although the investigators aimed to recruit PLWH who were poorly engaged in care and restricted eligibility to persons who were not taking ART and were not virally suppressed, most participants (80 %) nonetheless indicated on the anonymous questionnaire that they considered themselves to be in HIV care. Most of the 15 healthcare providers interviewed were physicians (80 %), in private practice (60 %), and nearly half (47 %) were ≥50 years old (Table 1b). Below we summarize the most frequent themes, provide illustrative quotes, and describe how the interview findings shaped the Data to Care program.

**Table 1:** Demographics of Qualitative Interview Respondents, Formative Work for the Care and Antiretroviral Promotion Program, Seattle, Washington, 2009

	Number	Percent
a. Persons Living with HIV/AIDS (*N* = 20)		
Gender & Sexual Orientation		
MSM	16	80
Male, sexual orientation unknown	1	5
Female	3	15
Age (years)		
<20	2	10
20–29	6	30
30–39	2	10
40–49	6	30
≥50	4	20
Race		
White	15	75
African American	1	5
Asian/Pacific Islander	1	5
Hispanic ethnicity	3	15
In HIV Medical Care	16	80
Last CD4 count (cells/mm^3^)		
<350	2	10
350–500	10	50
>500	8	40
b. HIV Care Providers (*N* = 15)		
Gender		
Male	9	60
Female	6	40
Age (years)		
30–39	4	27
40–49	4	27
≥50	7	47
Race		
White	13	87
African American	1	7
Asian/Pacific Islander	1	7
Practice Setting^a^		
Academic –Ryan White Part C	4	27
Academic –non-Ryan White	2	13
Non-academic – Ryan White	2	13
Private Practice	9	60
Credentials		
MD	12	80
PA or NP	3	20
Years in HIV Medical Practice		
<5	0	0
6–10	2	13
11–15	2	13
16–20	6	40
>20	3	20
Missing	2	13

^a^Categories are not mutually exclusive

### On the idea of the Health Department contacting individual PLWH to promote engagement in HIV care: what PLWH said

The majority of PLWH interviewed found it acceptable to be contacted by the health department to engage or re-engage them in HIV care. Two respondents commented:

*“Well, I think that would be a good thing, you know. Um, because not everybody’s all in the loop.”*

*“I think that’s a positive idea… having someone who’s grounded and in the business for a long period of time contacting me and asking me where I am, how I’m doing with it, I think it would bring me back to earth a little bit.”*

PLWH also emphasized that the health department could support PLWH in making choices affecting their health by providing information about HIV care and treatment. One respondent put it this way:

*“I think it’s good to always be informed, you know. I’d rather have too much information than not enough information, and I choose to make my decision based on, you know, the knowledge that I have.”*

Another respondent expressed appreciation for a proactive health department approach as part of the broader support system for PLWH when he said:

*“..if, you know, six months afterwards or even a year afterwards [after HIV diagnosis], I wasn’t getting, you know, in some kind of regime or something like that, it would be nice to know that, … the county’s here, looking and saying, ‘hey, you know, we don’t want you to fall through the cracks.”*

Two patients expressed the following concerns about health department-initiated contact:

*“I don’t know, it’s kind of an invasion, in a way….The doctor, your doctor, it’s his responsibility.”*

*“I don’t know why I have my kind of issues with health department people. It’s nothing personal, but it’s like, ‘how much information do I want to give the health department necessarily,’ because I don’t know what, why I’m doing it.”*

### On the idea of the Health Department contacting individual PLWH to promote engagement in HIV care: what HIV medical providers said

Medical providers had a range of opinions about the Data to Care concept, varying from supportive to neutral to negative. Some expressed concern about the program concept.

*“…I would have to be looking very carefully at the privacy issues there, patient choice issues…”*

*“… you are talking about crossing boundaries here between doctor-patient relationships.”*

*“I think it may actually be confusing if the public health messages and the provider messages are so different…um…that patients get confused and then don’t actually get the right care.”*

Another perspective was offered by providers who thought such a program could be a useful resource for their patients.

*“….the reality is that this is a person’s health. They should know what’s available to them.”*

*“..I think the patients can make their own decisions…I’m not opposed to more people knowing [about resources available to them to support HIV care].”*

*“If it’s an educational thing… I think that’s great. Because, as you know, provider time in a regular appointment is fairly limited and so it would be great if there were other avenues for education and why treatment is important and natural history of HIV.”*

Two providers’ comments suggested that the health department’s involvement in facilitating HIV care and treatment could fill a critical need for patients who had fallen out of HIV care.

*“Some people aren’t coming [to clinic] for anything and those people are really hard to get because we don’t have a means of getting to them, and providers are too busy and social work is too busy”*

*“Even though once they’ve lost insurance they’re technically not our patients, it’s too big of a public health risk to let them be out there just floating around without getting hooked up.”*

Some providers indicated that they would need more information to be convinced that health department outreach would add value to services already provided through an HIV clinic.

*“Would we reach out [to the health department] and say, ‘Look, we haven’t seen this guy in six months. Can you help us find him?’…I guess I’d have to know that it really worked. I’d have to see how good you were because we already have case managers, right?”*

When asked “Would it influence you to know what patients thought about it, as a provider, in how acceptable would it be?” one provider responded:

*“Yeah, absolutely. Absolutely, I mean, because if every patient said, ‘Yeah, no problem, I view it as simple education and someone’s looking out for me,’ then, yeah, I would definitely feel less concerned about it.”*

### Decisions about launching the program based on PLWH and provider input

Most respondents did not express opposition to the health department’s involvement in promoting HIV care and treatment, although some expressed hesitation based on concerns about patient privacy and the potential impact on the physician-patient relationship. Generally, PLWH and providers supported the idea that the health department could provide services that would complement those of case managers and medical providers. Most providers thought the health department could play an important role in systematically identifying out-of-care patients and reconnecting patients with medical care.

Based in part on the findings from these interviews, the health department enhanced the program’s emphasis on returning patients to HIV medical care and encouraging clients to continuously discuss ART initiation with their providers, emphasized to providers that DIS would not make treatment recommendations for individual patients, and incorporated procedures for sending letters to medical providers summarizing the encounters, if clients consented. To further address the concerns that some providers raised, the health department held additional meetings with providers to present the findings from the patient interviews and solicit feedback about the protocol to minimize conflict between the Data to Care program and individual patient-provider relationships.

### Structure of the program: comments from PLWH

The interviews helped PHSKC decide on a structure for the Data to Care program that would be acceptable to both providers and PLWH. Comments like the following from PLWH helped to shape the Data to Care program protocols for contacting PLWH by telephone.

*“I’ve been contacted before by different people from the public health…and it’s kind of, um, it can be a scary thing. So, you know, for me, I want to know why people are calling and I want to know, um…I don’t want to be left hanging, you know what I mean, with a phone call back, and you don’t know when it’s going to come.”*

Specifically, staff indicate early in the call and when leaving messages, that they are calling “about a program you are eligible for” to avoid the perception that the call is related to STD contact notification, and in messages, state when they will attempt to call again. Staff members also leave a phone number the client can call that leads directly to the Data to Care program counselor or, if the counselor is unavailable, a message with the counselor’s name and office hours.

When asked what type of information about HIV treatment would be useful to them, respondents expressed a need for plain language information about ART.

*“I would like it to be a little more colloquial. Something that I can actually absorb and something that is going to more like, ‘this is a drug, this is why you would take it, this is the effect that it is going to have on you.’… I have seen this god awful big poster with all drugs and I am like, ‘oh my god, this is intense’. I don’t want to be overwhelmed with gobbledygook… I just want to know, [A.] how is it going to affect me? and [B.] how much it is going to cost me?”*

*“…that’s what you should aim for: something that makes it understandable to people. ‘Cause it’s - we’re not pharmacists. Even the whole, uh, part about, you know, like it takes three different drugs and that sort of thing. It’s all like…I’ve got brain freeze! And so the simpler that you can make the basics, I think the better.”*

Several participants also described how hearing about the treatment experiences of HIV seropositive peers could have an impact different from that of health professionals.

*“…that would be nice to talk with someone who has been there and taken the meds…helping people who are just starting meds and talking to them about it and offering support. You know, ‘cause they’ve been there.”*

*“Hearing it from the horse’s mouth, just having the people who’ve, like, been there and done that.”*

*“Faces and testimonials are really important… if you can see a face of a real person to back up whatever it is you’re saying…examples of somebody’s experience… that’s a really positive thing. Because it’s like, ‘ah, yes, I’m not a number, I’m not a statistic, I’m a real person.’”*

Comments like these influenced the health department’s decision to implement the Data to Care program with a Data to Care program counselor who is skilled in conveying information about contemporary HIV treatment in an understandable way to diverse clients and who discloses his own HIV seropositive status to clients when relevant.

### Structure of the program: comments from providers

In general, providers emphasized the importance of the health department coordinating with provider and clinic-based efforts to engage patients in care and clearly delineating health department versus provider roles. Some expressed concern about the potential for duplication of efforts.

*“And I think, like I said earlier, supporting our case managers to do that type of thing. Because they know the patients, they know the environments they’re circulating in. It’s not threatening. If someone from the Health Department’s after you, well, the word gets around, and, you know, you’re gonna stay low.”*

Other providers expressed support for intentional redundancy, the health department working in parallel with clinic-based efforts to improve patient care engagement.

*“I think having a resource that’s larger than the individual clinic is huge….Yeah. I think the more [public health does] that works independent of the provider, the more likely you’ll be successful.”*

Many providers emphasized the need for the health department to help return patients to care.

*“And I think how public health resources would be better focused on the patients that we can’t get…. having better partnership with primary care providers for HIV to say, these are patients that were not – that are not following up. How could you help us with keeping them in care?”*

Almost all providers interviewed wanted to be informed about which of their patients the health department was attempting to contact and have the opportunity to be involved in the process. Providers suggested how the health department should approach persons contacted through a Data to Care Program.

*“I don’t know how you guys normally do it, but when you first talk to people, if they know this isn’t necessarily just because they have HIV -- that there are a number of infections that you contact people for -- that might kind of help normalize it.”*

*“I think maybe just being very forthright with the patient and explaining the importance of involvement in care, and trying to ascertain what it is about that unique individual that keeps him or her from seeking medical attention. Just asking, finding out what that barrier is, recognizing that it’s going to be different from one person to another.”*

### Decisions about structure of the program based on PLWH and providers’ input

PHSKC enhanced the involvement of medical providers in the program and designed the protocol to maximize coordination with the efforts of providers and case managers to keep patients involved in care. Specifically, the health department decided to notify providers before contacting their patients and send summaries after the encounter, if the client consents. The health department alerts to providers about which patients were eligible helped providers systematically monitor which of their patients had fallen out of care. In turn, this facilitated provider referrals to the Data to Care program and helped DIS obtain updated contact information for clients. PHSKC designed the DIS counseling session to focus on patient autonomy and right to informed decision-making while minimizing the potential to conflict with the providers’ advice. To this end, DIS educate program participants about contemporary ART and current treatment guidelines but avoid making individual recommendations.

After these interviews were completed, PHSKC HIV/STD program leaders and the disease intervention specialist who initially conducted the Data to Care outreach work presented the findings to HIV care providers and community representatives to discuss how the interviews had impacted the program design and solicit additional input. This process included a group meeting with community HIV providers, individual meetings with HIV providers in large volume clinics, and information distribution in a newsletter for HIV/AIDS sentinel providers; presentation to the King County HIV Prevention Planning Council and the UW Center for AIDS and STD Community Action Board; and presentation to two HIV case management groups.

Beginning approximately 1 year after the launch of the Data to Care program, the program leaders and DIS began periodically presenting findings from the program related to participant uptake and participant-reported barriers to care to the community in the form of presentations to the HIV Prevention Planning Council.

## Conclusions

U.S. health department HIV prevention leaders are faced with developing new relationships with the HIV healthcare system to achieve the common goal of improving the HIV care continuum. Our analysis highlights issues pertinent to the implementation of Data to Care programs. Generally, PLWH supported the concept of Data to Care and viewed the program as a source of additional support and information, while HIV care providers clearly indicated that health department outreach would be more acceptable if it were implemented in coordination with providers and case managers, to complement clinic-based efforts to improve patient engagement in HIV care.

The PHSKC program represents one of several possible Data to Care approaches; other health departments have described their experience with different models [6, 18–21]. The CDC distinguishes three types of Data to Care strategies, defined by who initiates contact with the patient: a health department model, a healthcare provider model, and a combination model. The Seattle-King County CAPP program is a combination model of Data to Care. As emphasized in the interviews, health department and clinic-based Data to Care approaches are not mutually exclusive. CDC and other U.S. Department of Health and Human Services agencies have emphasized the importance of cross-sector collaboration between health departments and clinical facilities [22–24]. Many PLWH who are inadequately engaged in HIV care have complex needs that cannot be addressed by one entity alone. Moreover, due to migration, unstable housing, and interruptions in cell phone service, many PLWH do not have stable contact information. Locating persons not connected to medical care requires multiple data sources and collaborative data sharing. In Seattle-King County, the health department Data to Care program shares information and coordinates with the patient outreach program located at the largest HIV clinic in the area [25].

The health department experience in Seattle-King County demonstrates that Data to Care programs can be successfully implemented with input from PLWH, HIV care providers, and other stakeholders. The program has been running for about 4 years with a combination of federal, state, and local funding. The environment of HIV care and prevention is markedly different today than it was at the time of these interviews in 2009–2010. The tension between treatment as prevention and treatment for individual health has dissolved, and CDC is encouraging the proactive use of HIV surveillance data to improve the HIV care continuum. Nonetheless, the concept of shared responsibility between health departments and HIV care providers to support lifelong engagement of PLWH in HIV medical care continues to gain traction [26]. Most U.S. state health departments are in the midst of working out the details of this collaboration. Community engagement remains vitally important for the success of Data to Care programs, and CDC recommends that health departments engage stakeholders both before launching a Data to Care Program and during the implementation and evaluation phases. Although in-depth individual interviews such as those we describe here may not be broadly feasible, all health departments can engage PLWH and providers through community meetings and outreach to key stakeholders.

Our study had several limitations. First, we did not track the response rate of persons who were offered interviews, nor did we collect more detailed patient characteristics, such as injection drug use. Our study was limited to one county and a small group of PLWH. Our findings may not reflect views of all PLWH and HIV providers in King County or other geographic areas. Finally, we conducted the interviews in 2009–2010, and although many of the themes addressed remain salient today, the views of the individuals interviewed may have changed since the time of the interviews.

In summary, the Data to Care strategy is an important component of public health efforts to improve the HIV care continuum. Health department contact directly with PLWH is feasible, but the acceptability and efficiency of this approach can be enhanced by involving medical providers in the process of identifying and contacting out-of-care patients and by coordinating with medical providers and case managers to assist patients in reengaging with HIV care and treatment.

## Abbreviations

ART, antiretroviral therapy; CAPP, Care and Antiretroviral Therapy (ART) Promotion Program; CDC, centers for disease control and prevention; DIS, disease intervention specialists; HIV, human immunodeficiency virus; PHSKC, Public Health – Seattle & King County; PLWH, persons living with HIV; STD, sexually transmitted disease; VL, viral load

## References

[1] Castel AD, Magnus M, Greenberg AE (2015). Update on the epidemiology and prevention of HIV/AIDS in the United States. Curr Epidemiol Rep.

[2] Greenberg AE, Purcell DW, Gordon CM, Barasky RJ, del Rio C (2015). Addressing the challenges of the HIV continuum of care in high-prevalence cities in the United States. J Acquir Immune Defic Syndr.

[3] Frieden TR, Das-Douglas M, Kellerman SE, Henning KJ (2005). Applying public health principles to the HIV epidemic. N Engl J Med.

[4] Fairchild AL, Bayer R (2011). HIV surveillance, public health, and clinical medicine--will the walls come tumbling down?. N Engl J Med.

[5] CDC. Data to Care: Improving Health and Prevention. 2015; https://effectiveinterventions.cdc.gov/en/HighImpactPrevention/PublicHealthStrategies/DatatoCare.aspx. Accessed 12 June 2015.

[6] Sweeny PA, Gardner LI, Buchacz K (2013). Shifting the paradigm: public health surveillance programs and HIV care providers collaborating to improve HIV care and prevent HIV infection. Milbank Q.

[7] CDC (2008). Recommendations for partner services programs for HIV infection, syphilis, gonorrhea, and chlamydial infection. MMWR.

[8] CDC (1995). Essential components of a tuberculosis prevention and control program. MMWR.

[9] Bayer R (1999). Clinical progress and the future of HIV exceptionalism. Arch Intern Med.

[10] Office of National AIDS Policy, The White House. The National HIV/AIDS Strategy: Updated to 2020. 2015; https://www.aids.gov/federal-resources/national-hiv-aids-strategy/nhas-update.pdf. Accessed 17 Aug 2015.

[11] Evans D, Van Gorder D, Morin SF, Steward WT, Gaffney S, Charlebois ED (2015). Acceptance of the use of HIV surveillance data for care engagement: national and local community perspectives. J Acquir Immune Defic Syndr.

[12] Dombrowski JC, Simoni JM, Katz DA, Golden MR (2015). Barriers to HIV care and treatment among participants in a public health HIV care relinkage program. AIDS Patient Care STDs.

[13] Buskin S, Kent JB, Dombrowski JC, Golden MR (2014). Migration distorts surveillance estimates of engagement in care: results of public health investigations of persons who appear to be out of HIV care. Sex Transm Dis.

[14] Carey JW, Gelaude D, Guest G, MacQueen KM (2008). Systematic methods for collecting and analyzing multidisciplinary team-based qualitative data. Handbook for team-based qualitative research.

[15] Panel on Antiretroviral Guidelines for Adults and Adolescents. Guidelines for the use of antiretroviral agents in HIV-1-infected adults and adolescents. Department of Health and Human Services. 2009; 1-161. Available at http://www.aidsinfo.nih.gov/ContentFiles/AdultandAdolescentGL.pdf. Accessed 7 June 2016.

[16] MacQueen K, McLellan-Lemal E, Bartholow K, Milstein B, Guest G, MacQueen KM (2008). Team-based codebook development: structure, process, and agreement. Handbook for team-based qualitative research.

[17] Hruschka DJ, Schwartz D, Cobb St. John D, Picone-DeCare E, Jenkins RA, Carey JW (2004). Reliability in coding open-ended data: Lessons learned from HIV behavioral research. Field Methods.

[18] Herwehe J, Wilbright W, Abrams A (2012). Implementation of an innovative, integrated electronic medical record (EMR) and public health information exchange for HIV/AIDS. J Am Med Inform Assoc.

[19] Griffin A, Jia Y, Samala R, Frison L, West-Ojo T, Kamanu-Elias N (2011). Recapture blitz in Washington DC: A model to strengthen HIV/AIDS surveillance and eliminate loss to follow up.

[20] Udeagu CC, Webster TR, Bocour A, Michel P, Shepard CW (2013). Lost or just not following up: public health effort to re-engage HIV-infected persons lost to follow-up into HIV medical care. AIDS.

[21] Buchacz K, Chen MJ, Parisi MK (2015). Using HIV surveillance registry data to re-link persons to care: the RSVP Project in San Francisco. PLoS One.

[22] Tailor AP (2014). 2014 Federal recommendations for HIV prevention services for persons with HIV: promoting synergies between clinicians, CBOs, and health departments.

[23] Insitute of Medicine (2012). Primary care and public health: exploring integration to improve population health.

[24] CDC (2015). National Center for HIV/AIDS, Viral Hepatitis, STD, and TB Prevention: Strategic Plan Through 2020.

[25] Bove J, Golden MR, Dhanireddy S, Harrington RD, Dombrowski JC (2015). Outcomes of a clinic-based, surveillance-informed intervention to relink patients to HIV care. J Acquir Immune Defic Syndr.

[26] Webster I (2015). Data to Care: From Start to Finish.

